# Epidemiological and Clinical Insights from 68 Veterinarian-Reported Cases of Feline Infectious Peritonitis During the Documented FIP Epizootic in Cyprus

**DOI:** 10.3390/pathogens15050499

**Published:** 2026-05-06

**Authors:** Demetris Epaminondas, Stella Mazeri, Maria Lyraki, Christine Tait-Burkard, Danielle Gunn-Moore, Stavroula Loukaidi, Efstathia-Evangelia Georgiadi, Stavros Loizides, Demetris Demetriou, Zoe Polizopoulou, Charalampos Attipa, Maria-Eleni Filippitzi

**Affiliations:** 1 Veterinary Services, Ministry of Agriculture, Rural Development and Environment, 1417 Nicosia, Cyprus; 2 Laboratory of Animal Production Economics, School of Veterinary Medicine, Aristotle University of Thessaloniki, 54124 Thessaloniki, Greece; 3 The Roslin Institute, Royal (Dick) School of Veterinary Studies, University of Edinburgh, Easter Bush, Midlothian EH25 9RG, UK; stella.mazeri@roslin.ed.ac.uk (S.M.); christine.burkard@roslin.ed.ac.uk (C.T.-B.); 4 Section of Small Animal Diseases, Faculty of Veterinary Medicine, The University of Veterinary Sciences Brno, 61242 Brno, Czech Republic; marialyraki@outlook.com; 5 Plakentia Veterinary Clinic, 17455 Athens, Greece; 6 Royal (Dick) Schooll of Veterinary Medicine Edinburgh, University of Edinburgh, Easter Bush, Midlothian EH25 9RG, UK; danielle.gunn-moore@ed.ac.uk (D.G.-M.); charalampos.attipa@ed.ac.uk (C.A.); 7 Vet Dia Gnosis Ltd., Stygos 27, 3117 Limassol, Cyprus; s.loukaidou@vetdiagnosiscy.com; 8 At the Vets Veterinary Clinic, Markou Evgenikou 10, 4002 Limassol, Cyprus; efstevagg@gmail.com; 9 D.S. Compass Solutions Ltd., Tamasou 12, Pano Deftera, 2460 Nicosia, Cyprus; sloizides@dscompass.eu (S.L.); ddemetriou@dscompass.eu (D.D.); 10 Diagnostic Laboratory, School of Veterinary Medicine, Aristotle University of Thessaloniki, 54627 Thessaloniki, Greece; poliz@vet.auth.gr; 11 Centre for Inflammation Research, Institute for Regeneration and Repair, The University of Edinburgh, Midlothian EH16 4UU, UK

**Keywords:** feline infectious peritonitis, FCoV-23, epizootic

## Abstract

In 2023, Cyprus experienced a large-scale epizootic of feline infectious peritonitis (FIP) temporally associated with the emergence of a novel feline coronavirus, FCoV-23. While molecular investigations have elucidated the recombinant origin of FCoV-23, field-based clinical and other epidemiological data from FIP cases reported during the epizootic period were needed to characterize the outbreak better. A prospective study was conducted using a structured 31-item questionnaire embedded in veterinary management software to characterize FIP cases diagnosed during the epizootic period (late 2022–2025). Data were voluntarily submitted by registered veterinarians across Cyprus. Cases were included based on a clinical diagnosis of FIP; virological confirmation of FCoV-23 infection was not required for inclusion. Data from 68 FIP cases reported by 22 clinics (response rate 21.0%) were analyzed. Affected cats were older than typically reported for FIP (mean age 3.9 years; median 3.0; range 0.4–12.9 years; SD 3.41). Most cases were documented in Limassol (51.5%) and Nicosia (25.0%). The most frequently reported clinical signs were non-specific, like anorexia (60.3%) and weight loss (54.4%), while a variety of neurological and mental manifestations were documented in 35.3% of cases. An albumin-to-globulin ratio < 0.8 was observed in 86.8% of tested cats. Antiviral therapy (GS-441524 or molnupiravir) was administered in 92.2% of cases, with reported clinical improvement of 88.9%. These findings demonstrate the value of questionnaire-based surveillance in documenting outbreak-associated FIP patterns. Although individual cases were not uniformly confirmed as FCoV-23 infections, the increased proportion of neurological presentations among FIP cases reported during the epizootic period supports previous molecular evidence suggesting that neurological involvement was associated with FCoV-23 circulation.

## 1. Introduction

Feline infectious peritonitis (FIP) is a fatal viral disease that is found worldwide [1,2]. To date, it remains one of the most important and challenging infectious diseases of cats, with significant clinical and epidemiological relevance worldwide. FIP is thought to be caused by virulent in-host mutated variants of feline coronavirus (FCoV) [3,4]. FCoV is part of the *Alphacoronavirus 1* species in the *Alphacoronavirus* genus of the *Coronaviridae* family [5,6,7]. Despite decades of study, its multifactorial pathogenesis, diverse clinical presentation, and high mortality continue to complicate diagnosis, prevention, and control [7,8,9]. Recent epizootic events, such as the large-scale outbreak reported in Cyprus in 2023 [10,11,12], have renewed scientific interest in the mechanisms of FCoV evolution and transmission, as well as in the conditions that may favor the emergence of highly pathogenic variants.

FCoV is highly prevalent in environments with multiple cats, with an infection rate as high as 90% where cats are housed together [13,14,15,16,17]. FCoV is distinguished into two pathotypes: the low-virulence Feline Enteric Coronavirus (FECV) and the highly pathogenic Feline Infectious Peritonitis Virus (FIPV), which causes FIP [4,5].

FCoV infection begins after oral ingestion of the virus, as the primary route of transmission is the fecal-oral route, usually through contact with contaminated cat litter, grooming, and fomites such as litter scoops or hairbrushes [18,19]. The virus replicates in the epithelial cells (enterocytes) of the small intestine, leading to viral shedding in feces [5]. Most FCoV infections remain subclinical, although mild enteritis may occur, especially where co-infections are present [4,20,21]. FIP is believed to develop due to the mutation of the less virulent FECV to a highly pathogenic FIPV in the host, which is known as the “internal mutation theory”. The mutation allows FIPV to target monocytes/macrophages, enabling its replication within circulating monocytes and macrophages, facilitating its systemic spread through these infected immune cells to the body tissues [5,22,23]. Clinically, this manifests as the effusive (or wet) form of FIP, characterized by fluid accumulation in the peritoneal, pleural, and/or pericardial cavities. In more chronic or localized cases, extensive perivascular pyogranulomatous lesions develop within affected organs. This is referred to as the non-effusive (or dry) form of FIP, with clinical signs depending on which organs are involved. Granuloma formation most commonly occurs in the spleen, liver, kidneys, intestines, heart, lungs, and eyes, while pyogranulomatous inflammation (vasculitis) may occur in the brain, spinal cord, and the meninges, resulting in a wide variety of clinical manifestations [18,24,25,26]. In addition, many cats develop clinical signs that are a combination of, or transition from, non-effusive to effusive disease [27,28,29,30,31].

The in-host mutation that enables the FIPV to actively replicate in the monocytes and macrophages can also affect transmission dynamics. More specifically, while FECV spreads primarily via the fecal-oral route, FIPV has generally limited transmission potential between cats as it rarely retains the capacity to replicate effectively within intestinal enterocytes [5]. Until recently, outbreaks of FIP that resulted from direct FIPV transmission were regarded as rare, isolated events [32,33] with limited epidemiological significance, reinforcing the prevailing belief that FIPV transmission between cats was minimal. This belief was changed when an outbreak of FIP was documented in 2023 in the island of Cyprus, a European Country in the Eastern Mediterranean Sea [10,11]. In the first half of 2023, the number of reverse transcription-polymerase chain reaction (RT-PCR)—confirmed FIP cases, based on samples from body cavity fluids, peritoneal lymph node aspirates, and tissue biopsies, rose dramatically, marking a more than 20-fold increase compared to 2022 [10]. Nevertheless, the cat population in Cyprus is predominantly composed of cared-for stray cats and shelter cats. As a result, only a small proportion of the affected cats that presented to the primary care practice had extensive diagnostics leading to RT-PCR confirmation [34]. Reports from the Pancyprian Veterinary Association (PVA) estimated that over 8000 cats were affected in the first half of 2023 [35]. The outbreak was found to involve a newly identified recombinant FCoV, named FCoV-23, which caused severe illness in both stray and domestic cats. Genetic analysis revealed that FCoV-23 had acquired a spike protein from a highly virulent canine coronavirus (CCoV), the Pantropic Canine Coronavirus (P-CCoV) [11,12,36]. The spike protein exchange observed in FCoV-23 is reminiscent of similar recombination mechanisms seen in other coronaviruses, such as SARS-CoV-2 [37], which have led to increased transmissibility and virulence [11,12].

Previous studies by our group have described the molecular features and geographic distribution of RT-PCR-confirmed FIP cases associated with the 2023–2024 epizootic in Cyprus [10,11,12,36]. However, systematically collected data from routine clinical practice, reflecting how cases were identified and presented to veterinarians during the outbreak, remain limited. The present study addresses this gap by analyzing prospectively collected data obtained through a structured questionnaire administered to veterinary clinics across Cyprus. By documenting clinician-reported FIP cases encountered in clinical settings during the epizootic, this study provides a complementary field-based perspective to existing laboratory and surveillance-focused investigations.

## 2. Materials and Methods

### 2.1. Data Collection

Beginning in March 2023, the Pancyprian Veterinary Association (PVA) contacted the authors to request guidance for the documentation and investigation of the ongoing FIP outbreak in Cyprus. Amongst other measures, the authors created a questionnaire (included as Supplementary Materials) which primarily aimed to document the characteristics of the FIP outbreak from the perspective of the practicing veterinarians. With the facilitation of the PVA an open invitation was extended to all registered companion animal or mixed practice veterinary clinics in Cyprus island-wide (this term refers to the areas that are under the effective control of the government of the Cyprus Republic), using the Vet Clinic Pro version 13.32 practice management system (D.S. Compass Solutions Ltd). The invitation encouraged veterinarians to contribute to data collection on FIP cases identified from 2022 onward. Veterinarians were contacted via an official email distributed by the PVA, which included comprehensive instructions, guidance documents, and study information. The data collection was managed through a structured questionnaire integrated into the veterinary management software Vet Clinic Pro version 13.32.

The questionnaire (included as Supplementary Materials) comprised 31 questions and was organized into three sections. The first section aimed to obtain information regarding the veterinary practice, including practice demographics and the unique identifier associated with the practice’s software management system. The second section aimed to capture epidemiological and background clinical information for each feline patient. Variables recorded included: patient age, sex, neutering status, breed, housing status (e.g., indoor/outdoor, multi-cat household), recent exposure to potential stressors, and reported contact with other cats, allowing characterization of individual and environmental-level factors relevant to disease occurrence. The third section aimed to document detailed clinical data, including observed clinical signs, results from laboratory and imaging investigations, confirmation of FIP by RT-PCR testing, and information on therapeutic interventions administered. Relevant diagnostic findings were entered directly into the questionnaire using data extracted from the patient’s electronic medical records via the same practice’s software management system.

To ensure the consistency and quality of data collection, the authors, with the facilitation of the PVA, organized a dedicated online seminar prior to the initiation of the questionnaire (May 2023). During this online seminar, the objectives of the data collection process were outlined, including an overview of the evolving outbreak and its epidemiological significance. At that time, the presentation focused on the documented increase in FIP cases, as information on the clinical manifestations of the outbreak was still limited. Comprehensive instructions were provided to participating veterinarians, detailing the procedures for accurate completion of the questionnaire and clarifying inclusion criteria for case identification. The online seminar also addressed best practices for clinical assessment and specimen collection to optimize diagnostic accuracy and data reliability. Veterinarians were encouraged to report cases from late 2022 onwards. All veterinarians were asked to read the consent declaration and indicate their agreement by ticking the consent box before completing the electronic questionnaire.

Case definitions were divided into three categories (A, B, and C):

Suspected cases were domestic feline patients with compatible clinical appearance [30] (e.g., loss of appetite, loss of weight, fever, peritoneal effusion, pleural effusion, pericardial effusion, ophthalmological and/or neurological signs), Category A cases. Those with additional compatible laboratory findings (e.g., hyperglobulinemia, hyperbilirubinemia, albumin to globulin ratio (A/G) < 0.5) were included as Category B cases.

Confirmed cases (Category C) were described by the reporting veterinarian as suspected cases with a positive FCoV RT-PCR result on a sample collected from either peritoneal effusion, pleural effusion, pericardial effusion, cerebrospinal fluid (CSF), aqueous fluid cell suspension, from fine needle aspiration biopsy (FNAB), or tissue biopsy. Additionally, positive cases were considered as those with positive FCoV immunohistochemistry on tissue biopsy with compatible pathological findings for FIP.

The case definitions and description were used to encourage the participating veterinarians to record data for all suspected cases, including ones that were not definitively confirmed. This is in line with the European Advisory Board of Cat Diseases (ABCD) FIP diagnostic tool algorithms [38] and is locally justified as PCR use for FIP diagnosis is limited.

For the purposes of this study, “clinical improvement” was defined as a veterinarian-reported positive response following initiation of treatment, based on resolution or marked improvement of presenting clinical signs (e.g., improved appetite, weight gain, resolution of effusions, improved mentation or neurological status), as recorded in the questionnaire. This assessment was purely based on the clinical judgment of the attending veterinarian.

### 2.2. Data Storage and Analysis

Data was securely stored on a server accessible exclusively to the software operator. Reports containing questionnaire responses, along with additional files and data, were exported in Microsoft Excel, version 2508 [39] format for analysis. Additional files—including blood tests and diagnostic imaging—were exported as individual files for each test, labeled with unique identifiers to ensure accurate patient tracking. Data analysis was performed using R Statistical Software version 4.4.2 [40]. Data manipulation, descriptive statistics, and visualization were performed using the tidyverse collection of packages, version 2.0.0 [41]. Given the descriptive nature of the study and the limited sample size, analyses were restricted to descriptive statistics. Continuous variables were summarized using mean, median, range, and standard deviation (SD), while categorical variables were summarized as counts and percentages. No inferential statistical tests were performed.

## 3. Results

### 3.1. Questionnaire Response

Out of 105 veterinary clinics contacted (105 veterinary clinics use the veterinary management software Vet Clinic Pro, version 13.32 out of 150 companion animal veterinary clinics island-wide in total), 22 responded, giving a response percentage of 21.0%. These clinics were spread across five districts in Cyprus: 10 in Limassol (45.5%), 6 in Nicosia (27.3%), 3 in Ammochostos (13.6%), 2 in Larnaca (9.0%), and 1 in Paphos (4.5%).

**Figure 1:**
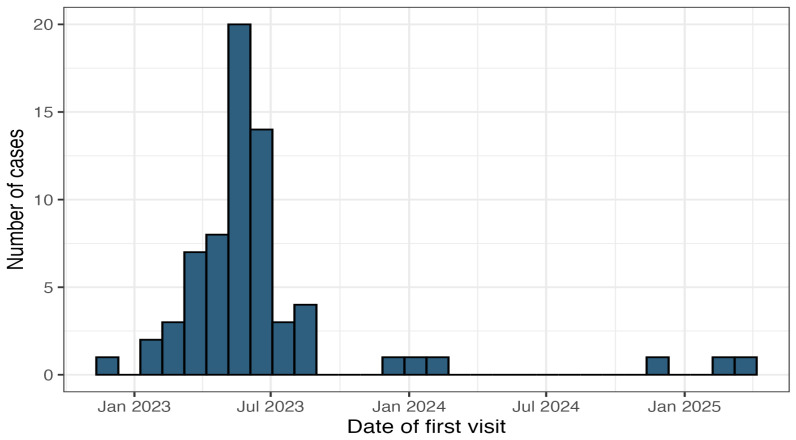
Timeline of the reported cases of feline infectious peritonitis (FIP) occurring in Cyprus between November 2022 and January 2025.

A total of 77 cases of FIP were reported. However, seven cases were excluded due to incomplete questionnaires, leaving 70 cases for analysis. Of these, two cases were also excluded as the reported occurrence was before the outbreak was declared (one case in February 2022 and another in July 2022), resulting in 68 cases for the final analysis. The cases covered the period from November 2022 to March 2025 (Figure 1), with the majority of the cases being reported between January 2023 and October 2023. Although the majority of the data were collected prospectively, nine cases were reported in the questionnaire at a different time from when the cases occurred.

The completeness of data varied across the many variables; however, most fields had low rates of missing data (<5%).

### 3.2. Case Signalment and Clinical History, Plus Geographical Distribution of Cases

The mean age of the cats in this population was 3.9 years (median = 3.0; min = 0.4, max = 12.9, SD = 3.41), indicating that most of the reported cases were young adults. Male cats accounted for 40/68 (58.8%) of the cases and female cats for 28/68 (41.2%). Most cats were neutered (55/68; 80.9%). The majority of cats were domestic short-hair (56/68; 82.4%), followed by domestic long-hair (10/68; 14.7%), while the British shorthair and Maine coon breeds represented one case each (1.5%). Regarding living environment, 30/68 (44.1%) cats lived both indoors and outdoors, 18/68 (26.5%) were kept exclusively outdoors, 12/68 (17.6%) were stray cats, and 8/68 (11.8%) were kept strictly indoors. The majority of cats (65/68; 95.6%) were reported to have contact with other cats; however, two of the indoor-only cats did not have contact with other cats. The third cat, which was declared not to have any contact with other cats, was reported as an indoor/outdoor cat. Over half of the cases originated from the Limassol district (35/68; 51.5%), followed by Nicosia (17/68; 25.0%), Ammochostos (10/68; 14.7%), then Larnaca and Paphos (3/68; 4.4% each) (Table A1).

### 3.3. Categorization and Clinical Presentation

Based on the classification scheme detailed in the Materials and Methods section, there were more Suspect cases (Category B) 40/68 (58.8%), followed by Confirmed cases (Category C) 15/68 (22.1%), then by Suspect cases (Category A) 13/68 (19.1%).

Among suspected cases without further laboratory confirmation (Category A; *n* = 13), Peritoneal effusion caused ascites was the most frequently observed clinical finding, reported in 10/13 cases (76.9%). Anorexia was recorded in 8/13 cats (61.5%), while neurological abnormalities, fever, weight loss, and reduced mental state were each documented in 4/13 cases (30.8%). Other clinical signs were infrequently observed in this category (Table 1).

The largest group of cases was diagnosed after clinical examination and in-house laboratory testing, without definitive confirmation by a RT-PCR (Category B; *n* = 40). In this group (Table 1), anorexia and weight loss were the most prevalent findings, each present in 24/40 cases (60.0%). Neurological signs were also commonly observed (17/40; 42.5%), followed by reduced mental state (12/40; 30.0%). Peritoneal effusion/ascites were identified in 14/40 cases (35.0%). Fever and ocular signs consistent with uveitis were each recorded in 8/40 cats (20.0%), while dyspnea attributable to pleural effusion and jaundice were observed in 7/40 cases each (17.5%). Hypothermia was uncommon, occurring in only 2 of 40 cases (5.0%).

Cases with molecular confirmation by RT-PCR (Category C; *n* = 15) exhibited a clinical presentation similar to that observed in Category B (Table 1). Anorexia and weight loss were again the most frequent clinical signs, each noted in 9/15 cases (60.0%). Peritoneal effusion/ascites were present in 5/15 cats (33.3%). Dyspnea due to pleural effusion was observed in 4/15 cases (26.7%), whereas neurological signs and reduced mental state were each documented in 3/15 cases (20.0%). Fever and jaundice were relatively uncommon, reported in 2/15 (13.3%) and 1/15 (6.7%) cases, respectively.

**Figure 2:**
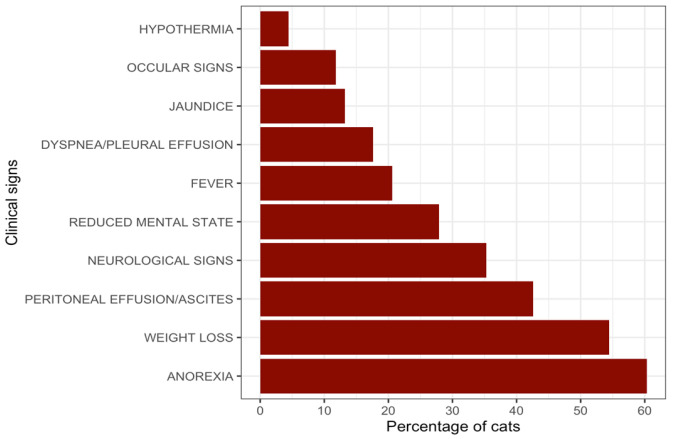
Graph presenting the clinical signs of FIP reported in this study’s cases (with the frequencies reported in percentages). The data were collected from the questionnaires submitted by participating veterinarians.

Across all cases (Suspected and Confirmed; Figure 2), the most frequent clinical signs were anorexia in 41/68 (60.3%) and weight loss in 37/68 (54.4%). Ascites was reported in 29/68 (42.6%), and neurological signs in 24/68 (35.3%). Additional findings included reduced mental state in 19/68 (27.9%), fever in 14/68 (20.6%), and dyspnea in 12/68 (17.6%). Jaundice was recorded in 9/68 (13.2%), ocular involvement (uveitis) in 8/68 (11.8%), and hypothermia in 3/68 (4.4%).

### 3.4. Co-Infections and Stressors

Of the cats tested for co-infections with an in-house Feline Leukemia Virus (FeLV) antigen test and Feline Immunodeficiency Virus (FIV) antibody test (36/68; 52.9%), all were FeLV negative. FIV positivity was detected in 5/36 (13.9%). The retrovirus status was unknown or untested in 32/68 (47.1%) of the cats. The only other co-infection reported was toxoplasmosis, which was reported to be positive by serology in 2/68 (2.9%) cats. Only one of those two cases was an FIP-confirmed RT-PCR (Category C) case. Most of the cats did not receive any pharmaceutical treatment at the time of their presentation (62/68; 91.2%) (Table A2).

A potential stress-related event prior to FIP-associated disease onset was reported in 14/68 (20.6%) of cats. This was most commonly a surgical procedure under general anesthesia (e.g., neutering; 6/14; 42.8%), followed by relocation (2/14; 14.3%), teeth cleaning (2/14; 14.3%), or other events (e.g., unspecified; 2/14; 14.3%), and travel or veterinary visits (1/14 each; 7.1%).

### 3.5. Hematology and Biochemistry

**Figure 3:**
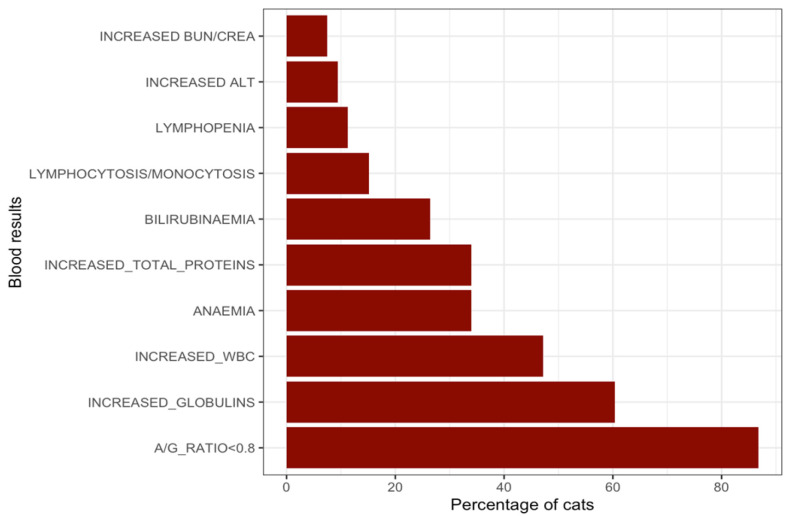
Graph presenting the whole blood and serum biochemistry results reported (with the frequencies reported in percentages). The data were collected from the questionnaires submitted by the participating veterinarians.

A total of 53 cases (suspected and confirmed; Figure 3) had hematology and/or serum biochemistry results included (but this had been performed by different point-of-care in-house analyzers). Among these, 46/53 (86.8%) showed an albumin-to-globulin (A/G) ratio < 0.8, making this the most consistent finding. Hyperglobulinemia was reported in 32/53 (60.4%) cases. Leukocytosis was present in 25/53 (47.2%), anemia in 18/53 (34.0%), and hyperproteinemia in 18/53 (34.0%). Hyperbilirubinemia was reported in 14/53 (26.4%), elevated Alanine Aminotransferase (ALT) in 5/53 (9.4%), and increased Blood Urea Nitrogen (BUN) and/or Creatinine in 4/53 (7.5%) (Table A3). Protein electrophoresis was performed in 31/68 (45.6%) cases, with the majority showing an increase in isolated gamma-globulins (24/31; 77.4%), followed by combined alpha- and gamma- increases (5/31; 16.1%), and isolated alpha increases (2/31; 6.5%) (Table A4). Among cases with increased gamma globulins, 19/24 (79.2%) had an A/G ratio < 0.8, and 7/31 (22.6%) had FIP confirmed by RT-PCR, including 4/31 (12.9%) from effusion fluid and 3/31 (9.7%) from FNAB. The combination of hyperglobulinemia and a low albumin-to-globulin (A/G) ratio was observed in 31 cases and varied according to clinical presentation. Among these, 11 cases (35.5%) had effusive-only ascites, 11 cases (35.5%) had neurological involvement, 5 cases (16.1%) were non-effusive, 2 cases (6.5%) had concurrent effusive and ocular involvement, and 2 cases (6.5%) presented with combined neurological and ocular involvement.

### 3.6. Diagnostic Imaging

In-house ultrasonographic examination was reported in 52/68 (76.5%) of cats; there were no abnormal findings in 32/68 of those (61.5%). Radiographs were available for 46/68 (67.6%) cases; abnormal findings were reported in 18/68 of those (39.1%) (Table A5).

### 3.7. FIP Molecular Testing, Confirmation of Diagnosis and Sequensing

A confirmed diagnosis of FIP by RT-PCR was obtained in 15 out of 68 cases (22.1%). Of these, sequencing was successfully performed for 14 cases, confirming them as FCoV23; one case, processed in a separate laboratory, lacked available sequencing data. Upon review, three of the fourteen RT-PCR FCoV23-confirmed cases matched entries in the database reported by Attipa et al. [11]. The remaining eleven cases underwent RT-PCR to ascertain spike domain 0 status and confirm FCoV-23, using primers 36A-F and 37A-R [11] and following the manufacturer’s instructions for VeriFi (PCRBio) polymerase. Sequence identity was established via Sanger sequencing. Of the samples tested with RT-PCR, 11 out of 68 cases (16.2%) were effusion samples, while 4 out of 68 cases (5.9%) were FNAB samples.

### 3.8. FIP Clinical Manifestation

**Figure 4:**
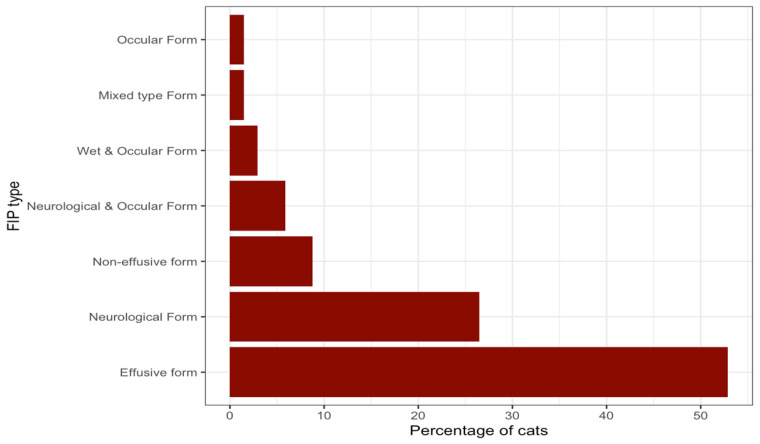
Graph presenting the clinical diagnosis of FIP reported as determined by the combination of clinical signs, clinical pathology, and diagnostic imaging findings reported in the questionnaires by the participating veterinarians (with the frequencies reported in percentages).

Based on clinical signs, clinical pathology, and diagnostic imaging findings documented by the participating veterinarians (all categories combined; Figure 4), the effusive form (thoracic, abdominal, and pericardial effusions) was the most frequently observed presentation, accounting for 36/68 (52.9%) cases. Predominant neurological signs were identified in 18/68 (26.5%) cases. The non-effusive form was less common, recorded in 6/68 (8.8%) cases. Combined neurological and ocular signs were reported in 4/68 (5.9%) cases, while concurrent effusive and ocular signs were reported in 2/68 (2.9%) cases. A mixed type (abdominal effusion and neurological and ocular involvement) presentation was reported in a single case (1.5%), as was a case with predominantly ocular signs (1.5%). Of the samples tested with RT-PCR, 11/68 (16.2%) originated from effusion samples, while 4/68 cases (5.9%) originated from FNAB samples.

### 3.9. Treatment and Outcome

Treatment was undertaken in 63/68 cases (92.3%), euthanasia was selected in 3/68 cases (4.4%), all belonging to Category A, and no treatment was recorded in 2/68 cases (2.9%). The most frequently reported therapy was oral GS-441524, administered in 33/63 treated cases (53.2%), followed by oral molnupiravir in 21/62 cases (33.3%), injectable remdesivir in 5/63 cases (7.9%), and a combination of injectable remdesivir with oral molnupiravir in 4/63 cases (6.3%). Most cases were treated with unlicensed products (43/63; 68.3%), whereas licensed formulations were used in 19/63 cases (30.2%).

Overall, a positive clinical response to treatment was reported in 56/62 cases (90.3%), while no clinical improvement was documented in 6/62 cases (9.7%). In Category A, all animals with documented treatment outcomes (9/9; 100%) exhibited a positive treatment response (Table 2). In Category B animals, a positive treatment outcome was recorded in 34/38; 89.5% and no response to treatment was recorded in 4/38; 10.5% (Table 2). In category C animals, a positive response was recorded in 13/15; 86.7% treatment was recorded, and no response to treatment was recorded in 2/15; 13.3% of the cases (Table 2). All cases treated with a combination of injectable remdesivir and oral molnupiravir demonstrated clinical improvement (4/4; 100%), as did all cats receiving remdesivir monotherapy (5/5; 100%). Among cats treated with oral GS-441524, a positive clinical response was observed in 30/33 cases (90.9%), whereas no response to treatment was reported in 3/33 cases (9.1%). For cases receiving oral molnupiravir monotherapy, clinical improvement was documented in 17/20 cases (85.0%), while 3/20 cases (15.0%) showed no response to treatment.

## 4. Discussion

The present study provides descriptive information on 68 cases that were diagnosed as FIP by qualified general-practice veterinarians during and after the documented 2023 outbreak in Cyprus [36]. These cases were documented using a standardized, structured questionnaire voluntarily completed by practicing veterinarians in real time as the outbreak evolved. It is important to emphasize that the present study was designed to capture field-based clinical observations rather than to function as a structured surveillance system with systematic molecular confirmation. A key methodological consideration is therefore the inclusion of cases classified as FIP based on clinical and diagnostic findings, without universal virological confirmation of FCoV-23 infection. This approach reflects the realities of veterinary clinical practice in Cyprus during the outbreak period, where access to RT-PCR testing was limited by cost, availability, and logistical constraints, particularly in a population that includes a large proportion of stray and unowned cats. As a result, diagnostic decision-making relied heavily on established clinical algorithms, incorporating signalment, characteristic clinical signs, and supportive laboratory abnormalities, such as hyperglobulinemia and a decreased albumin-to-globulin ratio, in line with European Advisory Board on Cat Diseases (ABCD) [30,38] recommendations. Importantly, the aim of this study was to document how FIP cases were recognized and managed in real-world clinical settings during an evolving epizootic, rather than to construct a strictly virologically confirmed case series. The inclusion of suspected cases (Categories A and B), therefore, provides a more representative account of the outbreak as experienced by veterinarians. However, this approach introduces the possibility of misclassification bias, as some cases may have represented other systemic diseases with overlapping clinical features. While response to treatment is a strong diagnostic indicator, which provides confidence in the inclusion of those cases, this limitation should be considered when interpreting findings, particularly those relating to clinical presentation and epidemiological patterns. Future studies incorporating systematic molecular confirmation would be necessary to validate and refine these observations.

The response rate of the study was 21.0% (22 clinics answered the questionnaire out of 105 contacted). Although modest, this response rate is comparable to those reported in questionnaire-based studies involving veterinary professionals, particularly in companion animal infectious disease research requiring detailed clinical data. Reported response rates in the veterinary literature vary widely depending on study design and target population [42,43,44,45], reflecting the practical challenges of achieving high participation in voluntary clinic-based surveys. Non-response bias cannot be excluded and should be considered when interpreting the results.

Most cases recorded in this study came from Limassol and Nicosia. This is not unexpected, as it reflects both the human population distribution as well as the cat population density [34] in these two districts of Cyprus. This finding may be affected by a potential bias due to the voluntary nature of participation, differences in veterinary clinic density, reporting practices, or engagement with the study, rather than true differences in disease incidence between the districts.

The typical signalment of cats with FIP includes young, non-pedigree cats, with a reported male predominance [26,29,31,46,47]. In the present dataset, the mean age of the 68 cats reported was 3.9 years. This is higher than the 1–2 years commonly reported in earlier literature [30,31], although age distributions have varied across the literature [10,11]. This finding is also in agreement with other FCoV23 studies [10,11,12,36]. One possible explanation for the relatively higher mean age observed in these 68 cases, particularly in the cases that coincide with the early phase of the outbreak [11,36], may relate to population-level susceptibility. At the onset of widespread viral circulation, cats across multiple age groups may have been immunologically naïve and therefore susceptible to developing clinical disease. Over time, shifts in exposure patterns or acquired immunity at the population level may have influenced the age distribution of reported cases, with proportionally more cases occurring in younger cats as the outbreak progressed. In contrast to reports indicating that pedigree cats account for up to 70% of typically described FIP cases [26,30,31], few pedigree cats were represented in the current dataset. This finding may reflect the demographic structure of the cat population in Cyprus, where large numbers of free-roaming and formerly unowned cats exist [34], and where the majority of owned cats are domestic shorthaired or domestic longhaired animals adopted from the streets [48]. The interpretation of breed and age distributions in this study is limited by the relatively small sample size and the underlying population structure of cats in Cyprus. The predominance of non-pedigree cats likely reflects the demographic composition of the local feline population rather than true breed predisposition. The overwhelming presence of cats with prior contact with other cats (95.6%) reinforces the recognized role of social and environmental transmission in FCoV maintenance [49]. This finding could also support the direct transmission dynamics of FCoV-23, which potentially caused the epizootic [10,11,12], but this study was not designed to directly answer this question. A proportion of cats (14/68; 20.6%) were reported to have experienced a stress-related event prior to the onset of clinical signs. This observation is consistent with previously suggested associations between stress and FIP development [50,51,52]. Overall, this study was not designed to assess risk factors associated with FCoV-23; these findings need to be interpreted with care.

The clinical manifestations recorded in this study were largely consistent with previous descriptions of FIP, including anorexia, weight loss, lethargy, and effusive peritoneal disease [26,29,31,53]. The notable rate of reported neurological signs (35.3%) deserves attention. This proportion exceeds the 10–30% range reported in earlier large population studies [26,30,31]. The neurological manifestation reported for this study reflects the diagnosis reached by participating veterinarians for each respective case, absent definitive confirmation. While this observation may indicate a change in clinical phenotype during the outbreak period, in agreement with the other FCoV-23 studies [10,11,12], it should be interpreted cautiously, due to the low number of confirmed FCoV-23 cases. Although all RT-PCR-confirmed and sequenced cases in this dataset were attributed to FCoV-23, the limited number of confirmed cases and the lack of systematic testing across all clinical presentations preclude definitive conclusions regarding altered tissue tropism or increased neurovirulence, as already proposed by a number of studies [54,55,56,57,58,59]. Furthermore, no direct comparative data from pre-epizootic periods are available within this study to determine whether the observed frequency represents a true shift in clinical phenotype or reflects increased awareness and reporting during the outbreak.

Diagnostic approaches reported by the veterinary practitioners on Cyprus reflected the practical challenges of field conditions. Only 22.1% (15/68) of cases were RT-PCR-confirmed as FIP, with most diagnoses being based on the combination of clinical signs and in-house laboratory test findings. A number of 14 out of the 15 RT-PCR confirmed FIP cases reported in this study were confirmed FCoV-23 through a second dedicated RT-PCR. The 15th sample was not available for further testing. Three of these cases belong to the sample pool reported by Attipa et al. [11,36], the publication that introduced FCoV-23.

These findings are consistent with patterns reported in several large clinical studies, where the cost and accessibility of molecular tests limit their use [17,26,60]. This situation is particularly evident in countries where companion animal health insurance systems are not established and where large populations of stray animals exist, such as in Cyprus [34,48], resulting in financial and logistical limitations that restrict the routine use of advanced diagnostic methods [61,62,63]. Despite this, most cases (55/68; 80.9%) included in-house analyzer blood examinations, reflecting the standard of animal care in Cyprus. The high prevalence of hyperglobulinemia and low serum albumin-to-globulin ratios (A/G < 0.8) mirrors typically observed FIP profiles and supports their continued diagnostic value [50]. A raised serum gamma-globulin concentration was the most common protein electrophoretic pattern (77.4%), consistent with the immunopathogenic nature of the disease [64,65,66]. Also, in line with the current literature is the finding that hyperglobulinemia and the A/G ratio are not dependent on the FIP form [26,30]. An additional noteworthy observation is that 14 out of 15 RT-PCR confirmed samples were identified as FCoV23. This supports Attipa et al.’s [11] claim that FCoV23 constitutes stable recombination, which caused an epizootic The consistent detection of FCoV23 over a span of more than two years also indicates its endemic presence within the feline population of Cyprus. This argument has to be supported by more evidence.

Therapeutically, Cyprus was the first European country to approve three different options for the treatment of FIP in cats: injectable Remdesivir (BOVA UK Ltd., 7-9 Gorst Road, Park Royal, London, NW10 6LA, UK), GS-441524 50mg tablets (BOVA UK Ltd., 7-9 Gorst Road, Park Royal, London, NW10 6LA, UK), and molnupiravir (Lagevrio™, Merck Sharp and Dohme Europe Inc. Boulevard du Souverain, 1170 Bruxelles, Belgium) [66]. The study confirms the widespread adoption of these antiviral drugs for the treatment of FIP, as 92% of the cases received one of these agents, or a combination, with an overall reported clinical improvement rate of 88.9%. These figures are consistent with published efficacy data of the antiviral drugs on FIP [25,28,31,66,67]. Treatment response rates differed across diagnostic categories. Notably, all Category A cases demonstrated a positive treatment response (9/9; 100%). In Category B, a high proportion of cases responded positively to antiviral therapy (34/38; 89.5%), while Category C also showed a substantial rate of positive response rate (13/15; 86.7%). Overall, the majority of treated cases (56/62; 90.3%) exhibited favorable outcomes, supporting the clinical judgment of the veterinarians involved. The high rate of reported clinical improvement following antiviral treatment in Categories A and B is consistent with the clinical suspicion of FIP in these cases. However, in the absence of confirmatory diagnostic testing, it cannot be definitively concluded that all such cases were correctly diagnosed. In particular, the small number of Category A cases and their reliance on clinical assessment alone introduces uncertainty, and the possibility of misclassification or spontaneous improvement in some cases cannot be excluded. Since 68.3% of the cats were given unlicensed preparations, concerns have to be raised regarding the legal and ethical issues surrounding the administration of such preparations [68,69]. Despite an expanding market for licensed treatments, unlicensed products are still widely used for FIP treatment [70]. While this study focuses on 68 specific cases, it is important to highlight that during the first six months of the outbreak in Cyprus, animal carers had no treatment options available. During this period, online providers of unlicensed products established an efficient distribution network, making these drugs easily accessible and limiting the use of licensed therapies, when the latter became available. This led the government of Cyprus to significantly reduce the cost of licensed molnupiravir once legal treatment options became available [65]. The information gathered for the licensed molnupiravir preparation referenced in the study (Lagevrio™, Merck Sharp and Dohme Europe Inc. Boulevard du Souverain, 1170 Bruxelles, Belgium) is useful for the knowledge of the use of this medication for the treatment of FIP in cats, which was specifically permitted for this use in Cyprus [65]. Administration of 25 cases, four of which were also administered with remdesivir, resulted in an 84% success rate. This outcome is consistent with studies showing high survival rates (78–92%) [71,72,73,74,75,76] for cats with FIP treated with generic molnupiravir, underscoring the drug’s effectiveness. The successful treatment of cats with FIP in Cyprus demonstrates the rapid dissemination of treatment protocols among veterinary practitioners during the outbreak. It exemplifies effective professional communication and adaptability in crisis conditions, as well as the crucial role the PVA played in crisis management. A major strength of this work lies in its prospective design, with almost real-time data collection. Once the outbreak was discovered, the authors, with the help of the PVA, asked veterinary practitioners on Cyprus to complete questionnaires for cases of FIP they had seen since the beginning of 2023. They were then asked to prospectively collect data for all new cases while the outbreak was ongoing; this minimized recall bias and allowed for a more accurate reflection of temporal and geographic trends. Unlike fully retrospective or laboratory-based studies, this approach provides a ground-level view of the dynamics in clinical practice. Moreover, the island-wide coverage achieved through the voluntary participation of veterinarians across all districts was something that occurred for the first time in Cyprus in the field of companion animals. Another key strength is the integration of clinical, epidemiological, and therapeutic data in one dataset. This allows a holistic interpretation of how the outbreak manifested, evolved, and was managed. Finally, this study demonstrates the feasibility and value of a questionnaire-based veterinary reporting model, which could be replicated for future similar events in companion animal medicine, especially where systematic surveillance programs do not exist. Furthermore, the findings of this study highlight the value of structured, questionnaire-based data collection as a practical and scalable approach for capturing real-world clinical information during emerging disease events. In particular, the integration of epidemiological, clinical, and therapeutic data provides useful insights into treatment practices and outcomes under field conditions.

Despite these strengths, several limitations must be acknowledged. The voluntary and self-reported nature of the questionnaire may have introduced selection bias: clinics that experienced more cases or were more aware of the outbreak may have been more likely to participate. If their diagnostic protocols differed from under-represented clinics, this could have biased the data. Second, diagnostic heterogeneity represents a significant limitation. The reliance on clinical and in-house diagnostic findings rather than molecular confirmation in most cases means that misclassification cannot be excluded. Although clinical patterns were strongly consistent with FIP, supported by positive response to FIP treatment in the majority of cases, some overlap with other systemic diseases is possible. An additional limitation of this study is the restricted sample size, which limits the ability to perform a formal evaluation of associations between risk factors such as signalment, stress-related events, and co-infections. As such, observations relating to these variables should be interpreted cautiously. Further studies are required to robustly investigate the effects of risk factors.

## 5. Conclusions

In summary, this study forms part of the broader investigation into the large-scale epizootic of FIP reported in Cyprus beginning in late 2022. The cases described here represent cats clinically assessed as FIP during the outbreak period. Compared with what is typically reported, the affected cats were older and predominantly non-pedigree. Neurological involvement was documented in 35% of cases, which is higher than the 10–30% commonly described in the earlier literature. Diagnostic approaches reported by veterinary practitioners reflected the practical constraints of field conditions. A minority of cases (15/68;22.1%) were confirmed by RT-PCR and (14/15;93%) further confirmed as FCoV-23, while most diagnoses were based on a combination of compatible clinical findings and in-house laboratory results (80.9%), consistent with routine clinical practice in Cyprus. Antiviral therapy was administered in the majority of cases (92.2%), primarily using a GS-441524 or molnupiravir (Lagevrio™), with clinical improvement reported in 88.9%. Overall, this study highlights the clinical patterns observed during the outbreak period and demonstrates the usefulness of structured questionnaires as a practical epidemiological tool for rapidly capturing real-time field observations during suspected emerging infectious disease events.

## Figures and Tables

**Table 1:** List of clinical exam findings listed by diagnostic category.

Clinical Signs	Overall (*n* = 68)	Category A (*n* = 13)	Category B (*n* = 40)	Category C (*n* = 15)
Abdominal Effusion/Ascites	29	10 (76.9%)	14 (35%)	5 (33.3%)
Anorexia	39	8 (61.5%)	24 (60%)	9 (60%)
Dyspnea/Pleural Effusion	41	1 (7.7%)	7 (17.5%)	4 (26.7%)
Fever	14	4 (30.8%)	8 (20%)	2 (13.3%)
Hypothermia	3	1 (7.7%)	2 (5%)	0 (0%)
Jaundice	9	1 (7.7%)	7 (17.5%)	1 (6.7%)
Neurological Signs	24	4 (30.8%)	17 (42.5%)	3 (20%)
Ocular Signs/Uveitis	8	0 (0%)	8 (20%)	0 (0%)
Reduced Mental State	19	4 (30.8%)	12 (30%)	3 (20%)

**Table 2:** Treatment response is stratified by diagnostic category.

Diagnostic Category	Response to Treatment	*n* = 62	Total Number	Percentage (%)
Category B	No Response	4	38	10.5
Category B	Positive Response to treatment with Clinical Improvement	34	38	89.5
Category A	Positive Response Clinical Improvement	9	9	100.0
Category C	No Response	2	15	13.3
Category C	Positive Response Clinical Improvement	13	15	86.7

## Data Availability

The data that support the findings of this study are available from the corresponding author upon reasonable request.

## Supplementary Materials

The following supporting information can be downloaded at: https://www.mdpi.com/article/10.3390/pathogens15050499/s1.

## Abbreviations

The following abbreviations are used in this manuscript:

FIP	Feline Infectious Peritonitis
FIV	Feline Immunodeficiency Virus
FeLV	Feline Leukemia Virus
CCoV	Canine Coronavirus
P-CCoV	Pantropic Canine Coronavirus
RNA	Ribonucleic acid
RT-PCR	Real-Time Polymerase Chain Reaction
ALT	Alanine Aminotransferase
BUN	Blood Urea Nitrogen
PVA	Pancyprian Veterinary Association

## Appendix A

**Table A1:** Table of results on case signalment and clinical history, plus geographical distribution of cases presented in Section 3.2.

Variable	*n* = 68	Percentage (%)
**Case Distribution Per District**		
Limassol	35	51.5
Nicosia	17	25.0
Ammochostos	10	14.7
Larnaca	3	4.4
Paphos	3	4.4
**Breed**		
Domestic Short-haired	56	82.4
Domestic Long-Haired	10	14.7
British Short-haired	1	1.5
Maine Coon	1	1.5
**Contact With Other Cats**		
Yes	65	95.6
No	3	4.4
**Living Environment**		
Indoor Outdoor	30	44.1
Outdoor only	18	26.5
Stray Cat	12	17.6
Strictly Indoor	8	11.8
**Neutering Status**		
Neutered	55	80.9
Intact	13	19.1
**Gender**		
Male	40	58.8
Female	28	41.2

**Table A2:** Table of results on co-infections presented in Section 3.4.

Variable	*n* = 68	Percentage
**Feline Leukemia Virus (FeLV) In-House Antigen Snap Test**		
Negative	36	52.9
Unknown or not performed	32	47.1
**Feline immunodeficiency virus (FIV) in-house antibody snap test**		
Unknown or not performed	32	47.1
Negative	31	45.6
Positive	5	7.4
**Other Co-Infections (Serology)**		
None	66	97.1
Toxoplasma Gondii	2	2.9

**Table A3:** Table of results for blood CBC and Biochemistry presented in Section 3.5.

Variable	*n* = 53	Percentage
Albumin to Globulin Ratio < 0.8	46	86.8
Elevated globulins	32	60.4
Elevated white blood cells	25	47.2
Anemia	18	34.0
Elevated Total Proteins	18	34.0
Bilirubinemia	14	26.4
Lymphocytosis/Monocytosis	8	15.1
Lymphopenia	6	11.3
Elevated Alanine Aminotransferase	5	9.4
Elevated Blood Urea Nitrogen/Creatinine	4	7.5

**Table A4:** Table of results on Serum Protein Electrophoresis presented in Section 3.5.

Variable	*n* = 31	Percentage
Protein Electrophoresis results		
increased gamma globulins	24	77.4
increased alpha and gamma globulins	5	16.1
increased alpha globulins	2	6.5

**Table A5:** Table of results on diagnostic imaging findings presented in Section 3.6.

Variable	*n* = 68	Percentage
**Ultrasound examination findings**		
No	32	47.1
Yes	20	29.4
Not performed	12	17.6
Missing data	4	5.9
**X-ray Findings**		
No	28	41.2
Yes	18	26.5
Not performed	17	25.0
Missing data	5	7.4
