# Supplementary material for: Epidemiological and Clinical Insights from 68 Veterinarian-Reported Cases of Feline Infectious Peritonitis During the Documented FIP Epizootic in Cyprus
Source: Pathogens. 2026 May 6;15(5):499. doi: 10.3390/pathogens15050499 (PMC13209701; doi:10.3390/pathogens15050499)
Supplement: Supplementary file 1 [file pathogens-15-00499-s001.zip › _3_MDPI_Viruses_1_supplementary_material_questionnaire.pdf]

# FIP QUESTIONNAIRE

Thank you for completing this questionnaire. Your participation is important for addressing the outbreak of feline infectious peritonitis (FIP) cases in Cyprus.

**Before completing the questionnaire, the veterinarian ticks the box on the consent part consent form available in Vet Clinic Pro.**

Please complete a separate questionnaire for each cat diagnosed with FIP. This questionnaire will be used for research purposes; however, no personal data that could identify the owner will be used. If you have any questions, please do not hesitate to contact us.

1. Please confirm that you are a veterinarian. If you are a cat owner of a cat with FIP, please ask your veterinarian to complete the survey.

   α. Veterinarian

   β. Non-Veterinarian

2. Name and postal code of the clinic where you work:

3. May we contact you if we require additional information about the case? If yes, please provide your name and a contact email:

4. Without stating the patient's name, please provide a unique identifier for the case (e.g. clinic internal ID, without owner or patient name):

5. District where the patient lives:

6. Has the patient travelled or originated from outside Cyprus? If yes, where:

7. Date of birth of the patient (DD/MM/YYYY, e.g. 24/03/2004):

8. Breed of the patient. If not purebred, please state DSH (Domestic Shorthair) or DLH (Domestic Longhair):

9. Sex of the patient:
a. Female intact
b. Female neutered
c. Male intact
d. Male neutered

10. Living environment:
a. Indoor only
b. Outdoor only
c. Indoor and outdoor
d. Stray

11. Does the patient have contact with other cats?
a. Yes
b. No

12. Have other cats in the same environment been diagnosed with FIP?
a. Yes
b. No

13. Body weight of the patient:

14. Body condition score:
a. 1–3 Underweight
b. 4–5 Ideal
c. 6–9 Overweight

15. Is the patient receiving treatment for other medical conditions? If yes, please specify medications (dose in mg/kg PO/IV/SC, SID/BID/TID):

16. Date of onset of clinical signs (DD/MM/YYYY):

17. Date of first presentation to the clinic (DD/MM/YYYY):

18. Briefly describe the patient's clinical signs:

19. Briefly describe the findings of the clinical examination:

20. Please upload the complete blood count and biochemistry results to Vet Clinic Pro.

21. FeLV antigen test result:
a. Positive
b. Negative
c. Not performed

22. FIV antibody test result:
a. Positive
b. Negative
c. Not performed

23. Diagnostic imaging findings (thoracic/abdominal ultrasound, radiographs, etc.):

24. Protein electrophoresis at diagnosis (specific test via Laboklin through Vet Dia Gnosis). Any remaining serum may be used for measurement of alpha-1-acid glycoprotein at no extra cost. The alpha-1-acid glycoprotein measurement is for research purposes only and results will not be available to the client.
a. Yes
b. No

25. How was the diagnosis of FIP established? (Note: serology and fecal tests are not diagnostic for FIP)
a. PCR positive on effusion
b. PCR positive on cytology or tissue biopsy
c. Positive immunohistochemistry / immunocytochemistry
d. No definitive diagnosis; strong suspicion based on clinical signs and history

26. If you answered a, b, or c, please upload the relevant results to Vet Clinic Pro.

27. Which form of FIP does the patient have?
a. Effusive (wet)
b. Non-effusive (dry)
c. Neurological
d. Ocular
e. Mixed – please specify: __________

28. What treatment has been initiated?
a. Remdesivir
b. GS-441524
c. Molnupiravir
d. Other drug – please specify: __________
e. Combination therapy – please specify: __________
f. No treatment

29. What dose was initiated? (mg/kg PO/IV/SC, SID/BID/TID):

| |
|---|
| |

30. If the patient is receiving antiviral therapy, what is the source of the medication?
a. Licensed products
b. Products of unknown origin via social media
c. Other – please specify: __________

The questionnaire has been successfully submitted.
